# The Impact of Donor-Recipient Weight Ratios and Body Surface Area Ratios on Outcomes After Adult Deceased Donor Whole-Liver Transplantation

**DOI:** 10.1097/TXD.0000000000001991

**Published:** 2026-08-21

**Authors:** Patrick B. McGeoghegan, John J. Miggins, Evert Sugarbaker, Diego Quirarte, Anirudha Karla, Tzu-Hao Lee, George Cholankeril, John A. Goss, Abbas Rana

**Affiliations:** 1 Department of Student Affairs, Baylor College of Medicine, Houston, TX.; 2 Division of Abdominal Transplantation, Michael E Debakey Department of Surgery, Baylor College of Medicine, Houston, TX.; 3 Section of Gastroenterology and Hepatology, Department of Medicine, Baylor College of Medicine, Houston, TX.

## Abstract

**Background.:**

The impact of donor-recipient size mismatch on deceased donor whole-liver transplant (DDLT) outcomes remains underexplored. Weight ratio (WR) is a simple, readily available size-matching metric, whereas body surface area ratio (BSAR) requires additional calculations. We analyzed the impact of donor-recipient WR and BSAR on outcomes among adult DDLT recipients and compared their predictive abilities.

**Methods.:**

We evaluated adult primary DDLTs in the United Network for Organ Sharing database from January 1, 2013, to January 1, 2024 (n = 69 511). Patients were divided into 5 WR and BSAR percentiles (<10th, 10th–20th, 20th–80th [reference], 80th–90th, and >90th). Cox models assessed associations with graft survival, patient survival, and length of stay (LOS). Kaplan-Meier and receiver operating characteristic (ROC) curves compared outcomes and predictive ability, respectively.

**Results.:**

WR and BSAR both impacted outcomes. Lower percentiles were associated with modestly decreased graft and patient survival. Graft survival was decreased in the <10th WR percentile (WR ≤0.65; hazard ratio [HR], 1.13; 95% confidence interval [CI], 1.06-1.20; *P* < 0.001), 10th–20th WR percentile (0.65< WR ≤0.74; HR, 1.06; 95% CI, 1.01-1.13; *P* = 0.03), <10th BSAR percentile (BSAR ≤0.78; HR, 1.14; 95% CI, 1.08-1.21; *P* < 0.001), and 10th–20th BSAR percentile (0.78< BSAR ≤0.85; HR, 1.10; 95% CI, 1.04-1.16; *P* = 0.001). Only the <10th WR percentile was associated with worse patient survival (HR, 1.08; 95% CI, 1.01-1.15; *P* = 0.02), whereas both the <10th BSAR percentile and 10th–20th BSAR percentile were associated (HR, 1.09; 95% CI, 1.02-1.16; *P* = 0.007; HR, 1.07; 95% CI, 1.01-1.14; *P* = 0.02, respectively). Both <10th and >90th WR and BSAR percentiles prolonged LOS. ROC analysis revealed identical areas under the ROC curve for WR and BSAR, suggesting similar predictive ability (c-statistic = 0.62).

**Conclusions.:**

WR and BSAR are both reasonable tools to assess donor-recipient size matching in DDLT. Lower size-matching ratios increased graft failure and mortality, whereas both extremes prolonged LOS. Given its simplicity and immediate availability during donor evaluation, WR may serve as a practical adjunct for donor-recipient size matching. Caution is warranted with WR ≤0.74 or BSAR ≤0.85.

## Graphical abstract

**Figure:**
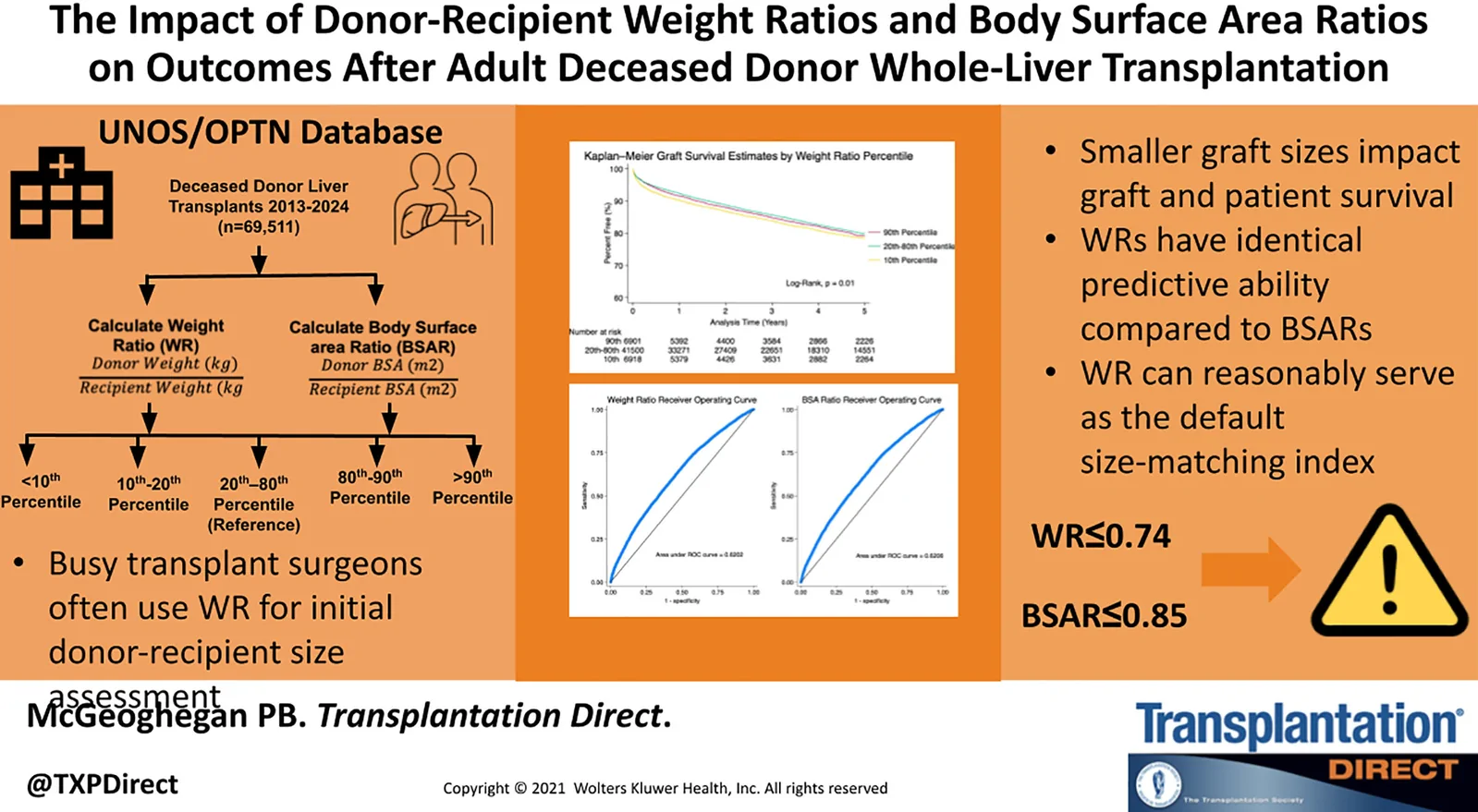

## INTRODUCTION

Optimal donor-recipient (DR) size matching is critical for graft function and patient outcomes across virtually all transplant domains.^1-4^ In liver transplantation, size mismatch contributes to small-for-size syndrome (SFSS) and large-for-size syndrome (LFSS). SFSS, mainly described in living donor liver transplantation (LDLT) literature, occurs when a donor graft is insufficient to maintain the metabolic and hemodynamic demands of the larger recipient, manifesting in cholestasis, portal hypertension, and death.^5,6^ LFSS, also mainly characterized in LDLT and pediatric deceased donor whole-liver transplant (DDLT) populations, occurs when a graft is too large for the recipient and is characterized by hypoperfusion from graft compression and inadequate flow from the recipient’s vessels. It leads to complications such as thrombosis, ischemia/reperfusion injury, delayed abdominal closure, and death.^7,8^

LDLT benefits from precise size-matching indices including standardized liver volume^9^ and graft-weight-to-recipient body weight ratio (WR).^10^ Because LDLT is an elective procedure, there is sufficient time to thoroughly assess DR size compatibility, often using 3-dimensional computed tomography to generate these indices. In contrast, DDLT is time-sensitive with multiple donor offers occurring each day and a narrow window for organ procurement. Therefore, size-matching methods must be both prompt and practical to ensure optimal DR pairing without delaying transplantation. In practice, DR body surface area ratio (BSAR) is often used and can be calculated from readily available donor and recipient anthropometric data.^11^ Some transplant surgeons contend that BSAR may better approximate organ size than WR because it is less affected by fluctuations in adipose tissue.^12,13^ However, WR offers several practical advantages. Donor and recipient weights are immediately available, allowing instantaneous assessment of size compatibility without additional calculations. Anecdotally, transplant surgeons today often make an initial assessment of DR size compatibility by comparing weights instead of BSAR, particularly in time-sensitive settings where multiple donor offers are evaluated in rapid succession. Despite the intuitive appeal of this approach, WR has not been rigorously evaluated as a size-matching metric in adult DDLT.

In pediatric DDLT, our prior analysis demonstrated that WR was comparable with BSAR in predicting transplant outcomes, with thresholds for poor outcomes at WR ≤0.70 and WR >2.0.^14^ Whether similar relationships exist in adult DDLT remains unknown. Therefore, it was necessary to explore and statistically validate WR as a size-matching metric in adult DDLT.

This study evaluates the impact of both WR and BSAR on transplant outcomes among adult DDLT recipients. We aim to determine whether 1 index is superior overall and to identify clinical scenarios in which one may be preferable to the other. We also analyzed subpopulations in which these ratios may have limited utility, including patients with at least moderate ascites.

## MATERIALS AND METHODS

### Study Population

We conducted a retrospective analysis using the United Network for Organ Sharing (UNOS) liver transplant registry, which includes data submitted to the Organ Procurement and Transplantation Network. Adult recipients (18 y or older) of primary whole-liver DDLT between January 1, 2013, and January 1, 2024, were included. Retransplants, combined or multivisceral transplants, LDLT, and split or partial grafts were excluded. Missing height or weight values at the time of transplant were also excluded (n = 55). Subgroup analysis was performed for recipients with the most severe small-for-size grafts (WR or BSAR <10th percentile), representing only a subset of grafts meeting the study definition of small-for-size (<20th percentile). This subgroup analysis was intentionally restricted to the most undersized grafts because any adverse outcomes observed in this population would be more likely attributable to size mismatch rather than other contributing clinical factors. Additional subgroup analysis was performed for patients with at least moderate ascites at transplant, as fluid overload from ascites may confound the relationship between size-matching ratios and outcomes. At least moderate ascites is defined by fluid accumulation that is noticeable on physical exam. Patients were followed from the time of transplant until death or most recent follow-up. This study does not qualify as human subjects research per US federal regulations and was exempt from Institutional Review Board review. As such, written informed consent was not required.

### Study Design and Variables

We evaluated the association between size mismatch and posttransplant outcomes, focusing on graft survival as the primary endpoint. Graft failure was defined as either death or need for retransplantation. Secondary outcomes included patient survival and length of stay (LOS). LOS was defined as days from transplant to discharge and was chosen because it is a corollary for perioperative complications. The primary predictors were WR and BSAR, defined as donor weight (kg) divided by recipient weight (kg) and donor BSA (m^2^) divided by recipient BSA (m^2^). BSA was calculated using the Mosteller equation (Equation 1).^15^


$$\begin{array}{c} \left(1\right):\;BSA\left(m^{2}\right)=\sqrt{\frac{\left[height\left(cm\right)\times weight\left(kg\right)\right]}{3600}} \end{array}$$

Patients were stratified into percentile-based categories for WR and BSAR^16^: <10th percentile, 10th–20th, 20th–80th (reference), 80th–90th, and >90th percentile. Percentile grouping allowed for evaluation of both undersized and oversized grafts while ensuring sufficient power for statistical comparisons. Small-for-size grafts were those with a WR or BSAR <20th percentile, whereas large-for-size grafts were those with WR and BSARs >80th percentile. The reference group was assumed to be the best-matched group by size. Donor and recipient variables were recorded at the time of DDLT.

### Statistical Analysis

Continuous variables are given as mean with SD, whereas categorical variables are shown as percentages. Percentile groups were compared via ANOVA for continuous variables and Pearson’s chi-square test for categorical variables. Chi-square tests were used for subgroup analysis.

A Cox proportional hazards model was constructed with variables at transplant chosen based on clinical experience and prior clinical scores,^17^ although some exploratory variables were chosen. Variables with a *P* value of <0.20 in the univariable analysis were retained in the model. Variables considered in the univariable analysis can be found in **Table S1** (**SDC,** https://links.lww.com/TXD/A892). We then evaluated the interaction between size matching and outcomes by separately adjusting for WR and BSAR for both donor and recipient variables. We also evaluated effect modification by selected covariates, incorporating interaction terms into our model. Specifically, we analyzed whether the association of size-matching ratios with outcomes differed between donation after circulatory death (DCD) and donation after brain death. Kaplan-Meier methodology estimated freedom from graft failure, death, and LOS. Log-rank tests assessed survival differences between curves. All results with a *P* value of <0.05 were considered significant.

To evaluate the predictive ability of WR and BSAR for 1-y graft failure, we generated receiver operating characteristic (ROC) curves adjusted for covariates. The area under the curve was calculated for each model to assess discriminative performance.

An exploratory analysis assessed whether WR and BSAR, when treated as continuous variables, satisfied the linearity assumption of the Cox proportional hazards model. To evaluate this, Martingale residuals from the Cox model were plotted against each predictor (WR and BSAR), and a locally weighted scatterplot smoothing (LOWESS) curve was overlaid. A linear pattern in the LOWESS curve suggests the assumption holds; nonlinearity indicates the assumption is violated. All statistical analyses were performed using Stata 18 (Stata Corp, College Station, TX).

## RESULTS

### Study Population

We identified 69 511 adult patients in the UNOS database who underwent whole-liver DDLT between January 1, 2013, and January 1, 2024. Among these patients, DR WR spanned from a minimum of 0.19 and a maximum of 3.9, whereas DR BSAR spanned from 0.32 to 2.2. WR percentile groupings were as follows: 0.19–0.65 (<10th percentile), 0.65–0.74 (10th–20th percentile), 0.74–1.2 (20th–80th percentile [reference]), 1.2–1.4 (80th–90th percentile), and 1.4–3.9 (>90th percentile). BSAR percentile groupings were the following: 0.32–0.78 (<10th percentile), 0.78–0.85 (10th–20th percentile), 0.85–1.1 (20th–80th percentile), 1.1–1.2 (80th–90th percentile), and 1.2–2.2 (>90th percentile).

Recipient and donor demographics and clinical characteristics stratified by DR WR are summarized in Table 1. The mean recipient age at transplant was 55.4 (±11.2) y, whereas the mean donor age was 42.7 (±15.9) y. Recipient BSA decreased with increasing DR WR, whereas donor BSA increased (*P* < 0.001). The mean recipient Model for End-Stage Liver Disease (MELD) score was 23.7 (±11.0), and recipients with WR ≤0.65 had an average MELD score of >25 (25.3 ± 11.5). Average cold ischemia time was 6.3 (± 2.8) h, and there were no significant differences between WR percentiles (*P* = 0.16).

**TABLE 1.** Demographic characteristics of recipients and donors stratified by bottom 10th, 10th–20th, 20th–80th, and upper 90th WR percentiles

	All recipients	% Entry completion	Recipients with WR≤0.65 (10th percentile)	Recipients with 0.65< WR ≤0.74 (10th–20th percentile)	Recipients with 0.74< WR ≤1.24 (20th–80th percentile)	Recipients with 1.2< WR ≤1.4 (80th–90th percentile)	Recipients with WR >1.4 (90th percentile)	*P*
N	69 511		6952	6949	41 707	6951	6952	
Recipient characteristics								
Age at transplant, y	55.4 (11.2)	100	54.1 (10.9)	55.2 (10.8)	55.7 (11.1)	55.5 (11.5)	55.2 (11.9)	**<0.001**
Height, cm	172.0 (10.3)	100	175.4 (10.4)	174.5 (10.0)	172.1 (10.1)	169.7 (10.0)	168.0 (10.2)	**<0.001**
Weight, kg	86.4 (20.4)	100	109.0 (22.6)	99.4 (18.0)	85.8 (16.9)	73.5 (14.2)	67.4 (13.9)	**<0.001**
BSA, m^2^	2.0 (0.3)	100	2.3 (0.3)	2.2 (0.2)	2.0 (0.2)	1.9 (0.2)	1.8 (0.2)	**<0.001**
Male	45 480 (65.4%)	100	4950 (71.2%)	4937 (71.0%)	27 756 (66.5%)	4119 (59.3%)	3718 (53.5%)	**<0.001**
Black	5198 (7.5%)	100	529 (7.6%)	525 (7.6%)	3122 (7.5%)	522 (7.5%)	500 (7.2%)	0.90
INR	2.0 (1.3)	100	2.1 (2.1)	2.1 (1.2)	2.0 (1.2)	2.0 (1.1)	2.0 (1.3)	**<0.001**
Creatinine, mg/dL	1.4 (1.1)	100	1.6 (1.3)	1.5 (1.1)	1.4 (1.1)	1.3 (1.0)	1.3 (0.9)	**<0.001**
MELD score	23.7 (11.0)	99.99	25.3 (11.5)	24.2 (11.2)	23.4 (11.0)	23.3 (10.6)	23.5 (10.4)	**<0.001**
Alcoholic hepatitis	896 (1.3%)	NA	107 (1.5%)	115 (1.7%)	523 (1.3%)	84 (1.2%)	67 (1.0%)	**0.002**
At least moderate ascites	23 305 (33.5%)	NA	2395 (34.5%)	2320 (33.4%)	13 688 (32.8%)	2345 (33.7%)	2557 (36.8%)	**<0.001**
Donor characteristics								
Age at transplant, y	42.7 (15.9)	100	39.2 (17.7)	41.7 (16.6)	43.1 (15.8)	43.7 (14.9)	43.7 (14.4)	**<0.001**
Height, cm	171.2 (10.7)	100	164.7 (13.1)	168.6 (10.4)	171.9 (10.1)	173.6 (9.9)	173.9 (9.9)	**<0.001**
Weight, kg	81.3 (21.3)	100	60.6 (13.3)	69.3 (12.6)	82.6 (16.6)	96.8 (18.8)	110.4 (23.9)	**<0.001**
BSA, m^2^	2.0 (0.3)	100	1.7 (0.2)	1.8 (0.2)	2.0 (0.2)	2.2 (0.2)	2.3 (0.3)	**<0.001**
Male	42 240 (60.8%)	100	3284 (47.2%)	3749 (54.0%)	26 291 (63.0%)	4605 (66.2%)	4311 (62.0%)	**<0.001**
Black	12 705 (18.3%)	100	1157 (16.6%)	1144 (16.5%)	7382 (17.7%)	1403 (20.2%)	1619 (23.3%)	**<0.001**
Bilirubin, mg/dL	0.85 (0.90)	99.99	0.8 (0.7)	0.8 (1.1)	0.9 (0.9)	0.9 (0.9)	0.8 (0.8)	**<0.001**
Creatinine, mg/dL	1.8 (2.0)	99.99	1.5 (1.8)	1.6 (1.8)	1.8 (1.9)	2.0 (2.1)	2.2 (2.2)	**<0.001**
Cold ischemia time, h	6.3 (2.8)	99.7	6.3 (2.8)	6.4 (2.8)	6.3 (2.8)	6.3 (2.8)	6.3 (2.9)	0.16

Bold numbers indicate *P* value of <0.05.

BSA, body surface area; INR, international normalized ratio; MELD, Model for End-Stage Liver Disease; NA, not applicable to this group; WR, weight ratio.

There are a larger proportion of male recipients in the smaller WR percentile groups, and there are a larger proportion of male donors in the larger WR percentile groups (*P* < 0.001). This suggests that there is a relationship between sex and size in adult DDLT. Additionally, 23 305 patients (33.5%) had at least moderate ascites at the time of transplant.

### Outcomes

Median time from transplant to either death, retransplant, or last follow-up was 3.2 y (interquartile range [IQR], 1.1–6.0). There were 1756 patients (2.5%) who underwent retransplant at a median of 2.5 (IQR, 0.39–14.3) mo.

### Graft Survival

The total time at risk for patients who were either censored or experienced graft failure was 272 617.41 patient-years. Overall, 13 936 patients (20.0%) experienced graft failure, 1756 (12.7%) of whom underwent retransplant, and 12 167 (87.3%) ultimately died. Overall graft survival was 92.0% (95% confidence interval [CI], 91.7-92.2) at 1 y posttransplant, and 79.5% (95% CI, 79.1-79.9) at 5 y.

**FIGURE 1.**
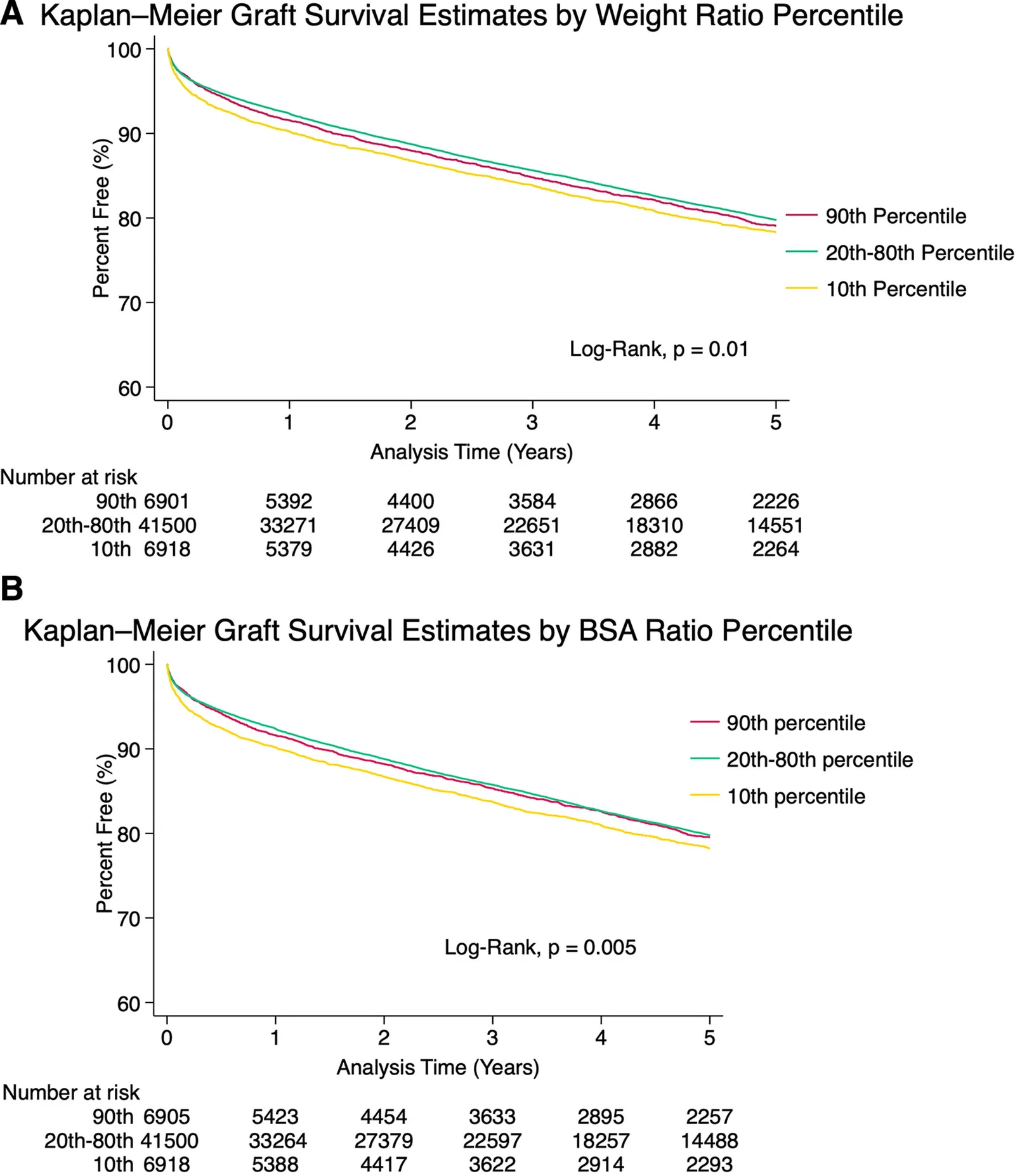
Graft survival based on weight ratio and BSA ratio percentiles. A, Kaplan-Meier estimate of graft survival stratified by <10th weight ratio percentile, 20th–80th weight ratio percentile (reference), and >90th weight ratio percentile. B, Kaplan-Meier estimate of graft survival stratified by <10th BSA ratio percentile, 20th–80th BSA ratio percentile (reference), and >90th BSA ratio percentile. BSA, body surface area.

When stratified by WR, small-for-size grafts, such as WR <0.65, had significantly decreased graft survival compared with the reference group (90.2% at 1 y and 78.3% at 5 y; Figure 1A; *P* = 0.01). Large-for-size grafts (WR >1.4) also exhibited lower rates of graft survival compared with the reference group but to a lesser degree (91.5% at 1 y and 79.1% at 5 y). BSAR percentiles followed similar trends throughout follow-up. BSAR ≤0.78 had reduced graft survival compared with the reference, with 1- and 5-y graft survival of 90.2% and 78.3%, respectively (Figure 1B; *P* = 0.005). One- and 5-y graft survival for BSAR >1.2 was 91.6% and 79.6%, respectively.

In multivariable analysis, the 2 lowest WR groups (WR <0.65 and 0.65< WR ≤0.74) had a significantly increased risk of graft failure (hazard ratio [HR], 1.13; 95% CI, 1.06-1.20; HR, 1.06; 95% CI, 1.01-1.13, respectively). Likewise, the 2 lowest BSAR groups (BSAR ≤0.78 and 0.78< BSAR ≤0.85) were associated with worse graft survival (HR, 1.14; 95% CI, 1.08-1.21, and HR, 1.10; 95% CI, 1.04-1.16, respectively). Larger graft sizes relative to the reference group were not associated with worse graft survival for either WR or BSAR (Table 2). Controlled DCD was also a significant predictor of decreased graft survival (HR, 1.38; 95% CI, 1.30-1.46; *P* < 0.001). However, interaction analysis showed that it did not modulate the impact of undersized grafts on graft survival. Specifically, the interaction between donor type (DCD versus donation after brain death) and either WR ≤0.74 or BSAR ≤0.85 was not significant (*P* > 0.05). We also explored the impact of graft size on discard rate by stratifying by donor weight and BSA. Smaller donors had lower discard rates compared with larger donors (**Table S2, SDC,** https://links.lww.com/TXD/A892).

**TABLE 2.** Multivariable model results for graft survival in all adult patients receiving deceased donor liver transplantation

Variable/graft failure	95% CI	*P*
WR percentiles		
Bottom 10th percentile (WR ≤0.65)	1.13 (1.06-1.20)	**<0.001**
10th–20th percentile (0.65< WR ≤0.74)	1.06 (1.01-1.13)	**0.03**
20th–80th percentile (0.74< WR ≤1.2)	Reference	
80th–90th percentile (1.2< WR ≤1.4)	0.99 (0.93-1.05)	0.70
Upper 90th percentile (WR >1.4)	1.03 (0.97-1.09)	0.32
BSAR percentiles		
Bottom 10th percentile (BSAR ≤0.78)	1.14 (1.08-1.21)	**<0.001**
10th–20th percentile (0.78< BSAR ≤0.85)	1.10 (1.04-1.16)	**0.001**
20th–80th percentile (0.85< BSAR ≤1.1)	Reference	
80th–90th percentile (1.1< BSAR ≤1.2)	1.00 (0.94-1.06)	0.96
Upper 90th percentile (BSAR >1.2)	0.99 (0.94-1.05)	0.78
ABO compatibility		
ABO incompatibility	1.08 (0.93-1.24)	0.31
ABO compatibility	1.05 (0.98-1.13)	0.20
Recipient age, y		
≤30	1.06 (0.95-1.17)	0.29
60–65	1.25 (1.19-1.30)	**<0.001**
≥65	1.44 (1.38-1.51)	**<0.001**
Donor age, y		
10–15	0.82 (0.69-0.96)	**0.01**
15–20	0.93 (0.86-0.99)	**0.03**
20–30	0.93 (0.88-0.97)	**0.002**
55–60	1.13 (1.07-1.20)	**<0.001**
60–70	1.21 (1.15-1.28)	**<0.001**
≥70	1.33 (1.22-1.44)	**<0.001**
Recipient albumin 1.5–2.0 mg/dL	1.12 (1.03-1.22)	**0.01**
Ascites	1.06 (1.01-1.11)	**0.01**
Spontaneous bacterial peritonitis	0.94 (0.89-1.00)	0.06
Recipient BMI ≥40 kg/m^2^	1.06 (0.98-1.14)	0.16
Cold ischemia time, h		
≤6	0.91 (0.88-0.94)	**<0.001**
≥14	1.10 (0.94-1.29)	0.22
Controlled donation after circulatory death	1.38 (1.30-1.46)	**<0.001**
Donor creatinine, mg/dL		
1.5–2.0	1.11 (1.05-1.18)	**<0.001**
≥2.0	1.10 (1.05-1.15)	**<0.001**
Hepatitis C cirrhosis	0.93 (0.88-0.97)	**0.003**
Hemodialysis	1.17 (1.10-1.25)	**<0.001**
Transplant center distance 500–1000 miles	1.01 (0.93-1.10)	0.80
Education		
High school	1.06 (1.02-1.10)	**0.004**
Bachelors	0.95 (0.90-1.00)	**0.04**
Doctorate	0.92 (0.85-0.99)	**0.02**
Hepatic encephalopathy	1.11 (1.05-1.16)	**<0.001**
Race		
Black recipient	1.21 (1.14-1.28)	**<0.001**
Black donor	1.06 (1.02-1.11)	**0.007**
Hepatocellular carcinoma	1.06 (1.00-1.11)	**0.033**
MELD score ≥40	1.03 (0.96-1.11)	0.40
Serum sodium, mEq/L		
125–130	0.95 (0.89-1.00)	0.056
≤125	0.96 (0.88-1.06)	0.43
145–150	1.12 (1.01-1.24)	**0.034**
150–155	1.23 (0.99-1.52)	0.06
155–160	1.70 (1.08-2.68)	**0.02**
KPS functional status		
10%	1.18 (1.07-1.30)	**0.001**
20%	1.12 (1.04-1.19)	**0.001**
50%	1.01 (0.95-1.07)	0.74
60%	0.89 (0.84-0.95)	**<0.001**
70%	0.89 (0.84-0.95)	**<0.001**
80%	0.84 (0.79-0.90)	**<0.001**
90%	0.75 (0.67-0.83)	**<0.001**
Recipient INR		
2.0–2.5	0.96 (0.91-1.01)	0.08
2.5–3.0	0.94 (0.88-1.00)	0.06
3.0–3.5	0.90 (0.83-0.98)	**0.01**
3.5–4.0	0.91 (0.80-1.02)	0.11
Clinical status		
Life support	1.11 (1.02-1.20)	**0.01**
ICU admission	1.08 (1.00-1.18)	0.06
Hospital admission	1.01 (0.95-1.07)	0.72
History of portal vein thrombosis	1.15 (1.08-1.22)	**<0.001**
Private health insurance	0.88 (0.84-0.91)	**<0.001**
Previous upper abdominal surgery	1.07 (1.03-1.11)	**<0.001**
OPTN region		
Region 1	1.09 (1.00-1.19)	**0.047**
Region 2	1.09 (1.03-1.16)	**0.002**
Region 3	0.96 (0.91-1.02)	0.17
Region 4	1.03 (0.97-1.10)	0.28
Region 5	0.89 (0.84-0.95)	**<0.001**
Region 6	0.85 (0.76-0.95)	**0.004**
Region 10	0.94 (0.88-1.00)	0.06
Donor ALT ≥100 IU/L	0.98 (0.94-1.02)	0.34
National allocation	1.08 (1.02-1.15)	**0.005**
Recipient total bilirubin		
≤2 mg/dL	1.16 (1.11-1.22)	**<0.001**
8–16 mg/dL	0.99 (0.94-1.05)	0.82
TIPS procedure	1.09 (1.03-1.15)	**0.002**

Bold numbers indicate *P* value of <0.05.

ALT, alanine aminotransferase; BMI, body mass index; BSAR, body surface area ratio; CI, confidence interval; ICU, intensive care unit; INR, international normalized ratio; KPS, Karnofsky Performance Status; MELD, Model for End-Stage Liver Disease; OPTN, Organ Procurement and Transplantation Network; TIPS, transjugular intrahepatic portosystemic shunt.

### Patient Survival and LOS

**FIGURE 2.**
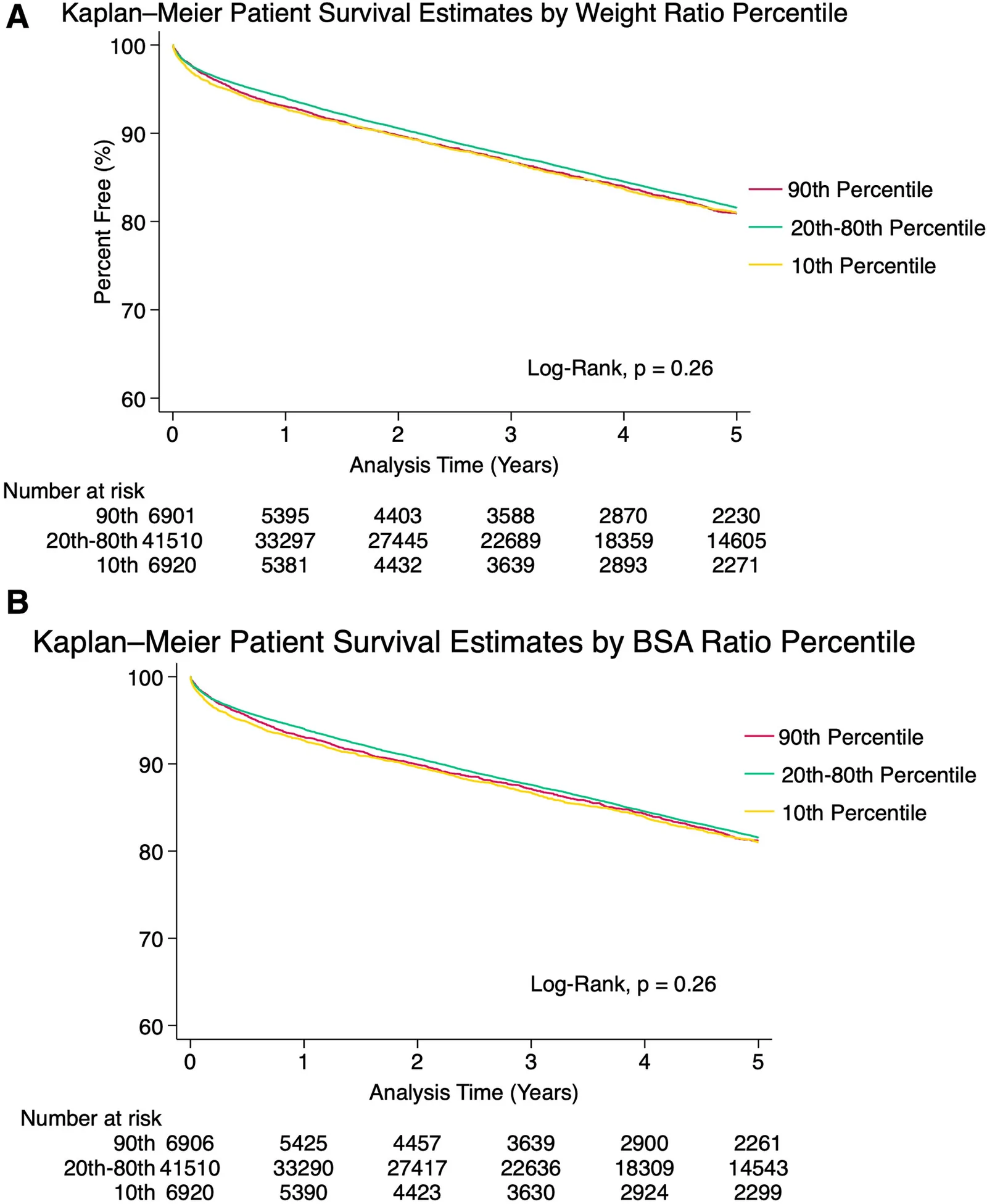
Patient survival estimates based on weight ratio and BSA ratio percentiles. A, Kaplan-Meier estimate of patient survival stratified by <10th weight ratio percentile, 20th–80th weight ratio percentile (reference), and >90th weight ratio percentile. B, Kaplan-Meier estimate of patient survival stratified by <10th BSA ratio percentile, 20th–80th BSA ratio percentile (reference), and >90th BSA ratio percentile. BSA, body surface area.

There were 12 696 (18.3%) deaths during the study period, and the total time at risk was 273 206.12 patient-years. Overall patient survival was 93.7% (95% CI, 93.5-93.9) at 1 y posttransplant and 81.4% (95% CI, 81.1-81.8) at 5 y posttransplant. When stratified by WR percentile, patient survival was lower than the reference value among both large and small graft sizes (WR ≤0.65 and WR >1.4), although not to a significant degree (*P* = 0.26; Figure 2A). The same trends are held for BSAR (BSAR <0.78 and BSAR >1.2; *P* = 0.26; Figure 2B).

When adjusted for other factors in the model, only WR <0.65 was associated with worse patient survival (HR, 1.08; 95% CI, 1.01-1.15; *P* = 0.02). In contrast, both BSAR ≤0.78 (HR, 1.14; 95% CI, 1.08-1.21) and 0.78< BSAR ≤0.85 (HR, 1.10; 95% CI, 1.04-1.16) were associated with worse patient survival (**Table S3, SDC,** https://links.lww.com/TXD/A892).

**FIGURE 3.**
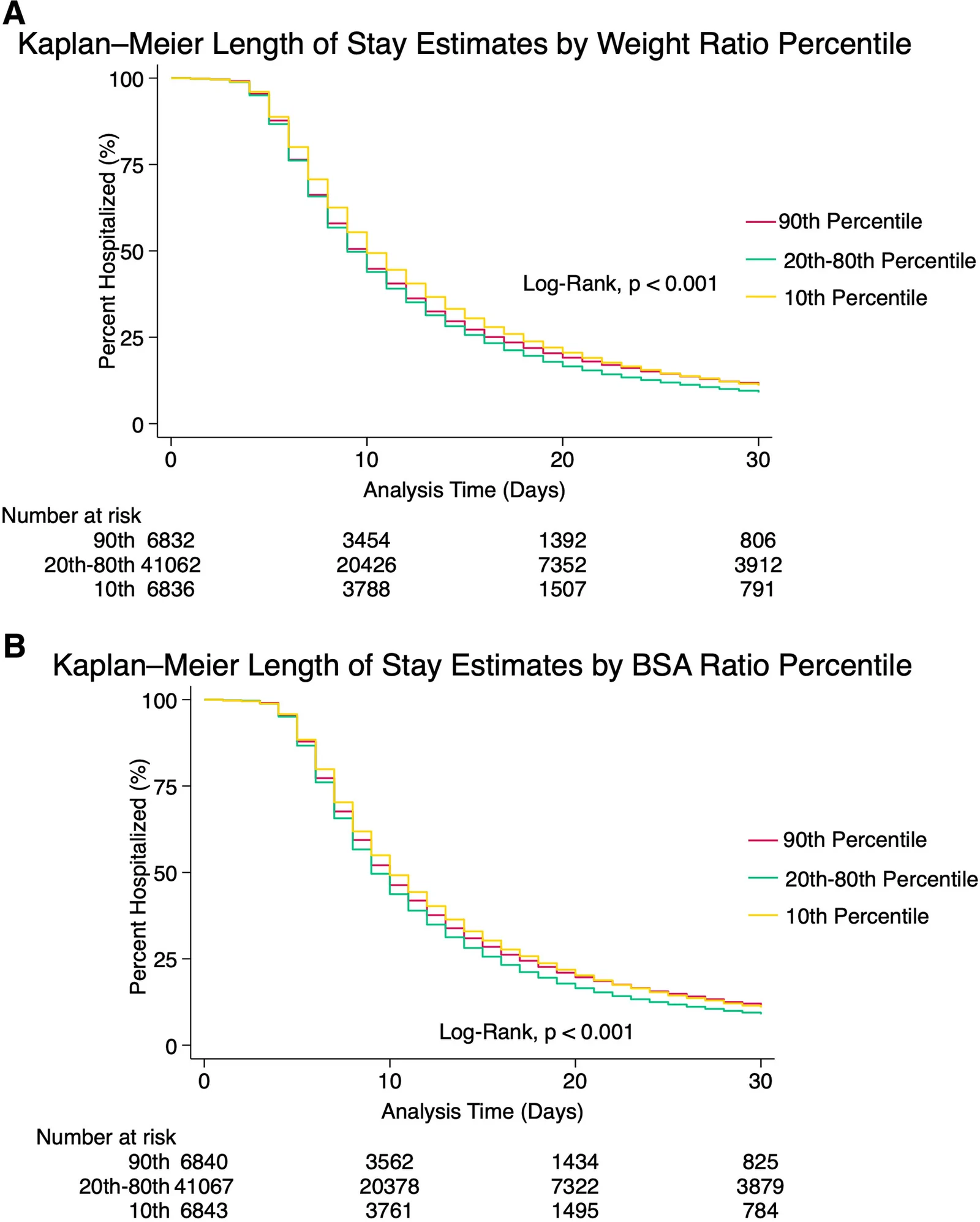
Length of stay estimates by weight ratio and BSA ratio percentiles. A, Kaplan-Meier estimate of length of stay stratified by <10th weight ratio percentile, 20th–80th weight ratio percentile (reference), and >90th weight ratio percentile. B, Kaplan-Meier estimate of length of stay stratified by <10th BSA ratio percentile, 20th–80th BSA ratio percentile (reference), and >90th BSA ratio percentile.

The median LOS among all patients was 10 d (IQR, 7–16 d). The median LOS among patients with WR ≤0.65 was 10 d (IQR, 7–17 d) compared with 9 d (IQR, 7–16 d) for the reference group (20th–80th percentile). The same values held for patients with BSAR ≤0.78. The median LOS for patients with WR >1.4 (and BSAR >1.2) was 10 d (IQR, 7–18 d). Kaplan-Meier curves show higher hospitalization rates over follow-up for smaller graft sizes (WR ≤0.65 or BSAR ≤0.78). Larger graft sizes (WR >1.4 and BSAR >1.2) also had elevated hospitalization rates compared with reference but not to the same degree as smaller graft sizes (Figure 3).

In multivariable analysis, both extremes of WR and BSAR were associated with prolonged LOS. Patients with WR ≤0.65 (HR, 0.95; 95% CI, 0.92-0.97; *P* < 0.001) and BSAR ≤0.78 (HR, 0.94; 95% CI, 0.92-0.97; *P* < 0.001) were at risk of prolonged hospitalization. Patients with WR >1.4 (HR, 0.94; 95% CI, 0.92-0.97; *P* < 0.001) and BSAR >1.2 (HR, 0.94; 95% CI, 0.91-0.96; *P* < 0.001) were also at risk of prolonged hospitalization (**Table S4, SDC,** https://links.lww.com/TXD/A892).

### Subgroup Analysis: Small-for-size Grafts

Among patients with small-for-size grafts, there were 1253 patients who experienced graft failure (21.0%) and 1097 (18.4%) died. Advanced donor and recipient age, DCD, increased donor creatinine, and lower functional status were all associated with increased graft failure, whereas shorter cold ischemia times as well as recipient and donor hepatitis C virus (HCV) had a protective effect (likely due to the fact that HCV grafts tend to be systematically younger and higher quality and that HCV cirrhotics are transplanted at lower average MELD scores; Table 3).

**TABLE 3.** Adjusted multivariable results for graft survival among patients with small-for-size grafts

Variables	HR (95% CI)	*P*
ABO incompatibility	1.42 (0.89-2.27)	0.147
Recipient age, y		
60–65	1.36 (1.18-1.57)	**<0.001**
≥65	1.33 (1.13-1.57)	**0.001**
Donor age, y		
≤10	0.84 (0.62-1.13)	0.247
10–15	0.77 (0.57-1.04)	0.087
15–20	1.16 (0.96-1.41)	0.133
20–30	0.89 (0.76-1.05)	0.17
55–60	1.22 (1.00-1.49)	0.052
60–70	1.22 (1.01-1.47)	**0.043**
≥70	1.80 (1.37-2.36)	**<0.001**
Recipient BMI, kg/m^2^		
35–40	0.91 (0.79-1.05)	0.194
≥40	1.10 (0.95-1.28)	0.204
Cold ischemia time, h		
≤6	0.85 (0.76-0.95)	**0.006**
12–14	1.49 (0.94-2.36)	0.092
≥14	0.53 (0.27-1.03)	0.063
Controlled donation after circulatory death	1.61 (1.33-1.94)	**<0.001**
Donor creatinine ≥2.0 mg/dL	1.23 (1.06-1.42)	**0.007**
Hepatitis C cirrhosis	0.81 (0.67-0.98)	**0.03**
Hemodialysis	1.10 (0.90-1.35)	0.366
Transplant center distance ≥1000 miles	0.52 (0.29-0.93)	0.028
Education		
High school	1.10 (0.98-1.24)	0.111
Doctorate	0.89 (0.70-1.12)	0.313
Hepatic encephalopathy	1.04 (0.88-1.24)	0.64
Black recipient	1.29 (1.06-1.56)	**0.009**
MELD score		
30–35	0.96 (0.79-1.17)	0.674
≥40	1.03 (0.84-1.26)	0.796
Serum sodium ≤120 mEq/L	0.77 (0.54-1.10)	0.145
KPS functional status		
10%	1.38 (1.02-1.85)	**0.034**
20%	1.11 (0.90-1.36)	0.339
90%	0.78 (0.55-1.09)	0.148
HCV-positive serology (donor)	0.71 (0.55-0.91)	**0.007**
INR 2.0–2.5	0.88 (0.75-1.03)	0.123
Clinical status		
Life support	1.11 (0.87-1.41)	0.41
ICU admission	1.23 (0.96-1.59)	0.105
Hospital admission	1.07 (0.89-1.27)	0.488
Private health insurance	0.84 (0.74-0.95)	**0.005**
OPTN region		
Region 3	1.16 (0.99-1.36)	0.058
Region 4	1.19 (0.99-1.42)	0.061
Region 6	0.88 (0.61-1.26)	0.485
Region 8	0.78 (0.59-1.02)	0.074
Region 10	0.82 (0.66-1.02)	0.077
Recipient total bilirubin ≤2 mg/dL	1.22 (1.05-1.42)	**0.008**

Bold numbers indicate *P* value of <0.05.

BMI, body mass index; CI, confidence interval; HCV, hepatitis C virus; HR, hazard ratio; ICU, intensive care unit; INR, international normalized ratio; KPS, Karnofsky Performance Status; MELD, Model for End-Stage Liver Disease; OPTN, Organ Procurement and Transplantation Network.

### Subgroup Analysis: Ascites

Of the 23 305 patients with at least moderate ascites, 4557 (19.6%) experienced graft failure, and there were 4202 (18.0%) deaths. Adjusted analyses for patients with at least moderate ascites (n = 23 305) demonstrated that a WR ≤0.64 (<10th WR percentile) was associated with decreased graft survival (HR, 1.11; 95% CI, 1.00-1.22; *P* = 0.04). Both BSAR ≤0.78 (<10th BSAR percentile) and 0.78< BSAR ≤0.85 (10th–20th BSAR percentile) were associated with reduced graft survival (HR, 1.15; 95% CI, 1.05-1.27, and HR, 1.11; 95% CI, 1.01-1.23, respectively; Table 4). Kaplan-Meier graft survival curves show lower WR and BSAR percentiles with decreased graft survival compared with the reference, although not to a statistically significant level (log-rank; *P* > 0.05; **Figure S1A and B, SDC,** https://links.lww.com/TXD/A892).

**TABLE 4.** Adjusted multivariable results for graft survival in patients with at least moderate ascites receiving deceased donor liver transplantation

	HR (95% CI)	*P*
WR percentiles		
Bottom 10th percentile (WR ≤0.64)	1.11 (1.00-1.22)	**0.04**
10th–20th percentile (0.64< WR ≤0.74)	1.09 (0.99-1.21)	0.07
20th–80th percentile (0.74< WR ≤1.3)	Reference	
80th–90th percentile (1.3< WR ≤1.4)	0.99 (0.90-1.10)	0.92
Upper 90th percentile (WR >1.4)	1.01 (0.91-1.12)	0.83
BSAR percentiles		
Bottom 10th percentile (BSAR ≤0.78)	1.15 (1.05-1.27)	**0.004**
10th–20th percentile (0.78< BSAR ≤0.85)	1.11 (1.01-1.23)	**0.03**
20th–80th percentile (0.85< BSAR ≤1.1)	Reference	
80th–90th percentile (1.1< BSAR ≤1.2)	0.99 (0.89-1.09)	0.79
Upper 90th percentile (BSAR >1.2)	0.98 (0.89-1.09)	0.77

Covariates used in each model are not included in the table. Bold numbers indicate *P* value of <0.05.

BSAR, body surface area ratio; CI, confidence interval; HR, hazard ratio; WR, weight ratio.

### WR and BSAR: Comparing Predictive Ability and Assessing Linearity

**FIGURE 4.**
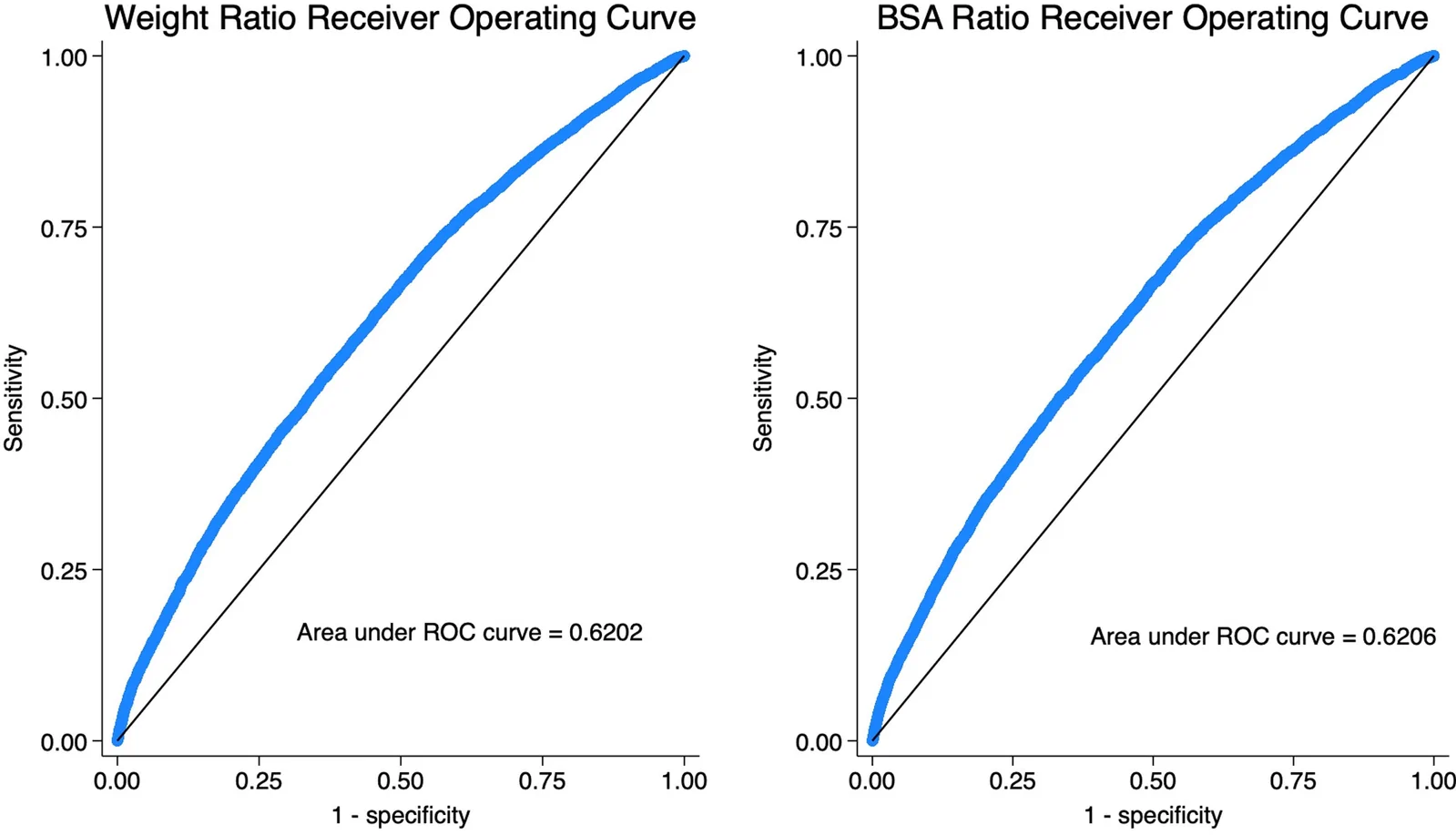
ROC curves with areas under the curve comparing the predictive ability of multivariable logistic regression models constructed for weight ratio and BSA ratio for 1-y graft survival. BSA, body surface area; ROC, receiver operating characteristic.

The areas under the ROC curve for graft failure at 1 y were identical in adjusted multivariable models for both WR and BSAR (c-statistic = 0.62; Figure 4). As Cox regression assumes a direct relationship between dependent and independent variables, we used Martingale residuals to evaluate whether continuous WR and BSAR variables violate the linearity assumption. LOWESS functions appeared nonlinear, with residuals reaching a minimum of zero at WR 1 and BSAR 1 (**Figure S2A and B, SDC,** https://links.lww.com/TXD/A892).

## DISCUSSION

Our study demonstrated that high and low WR and BSAR were associated with worse posttransplant outcomes in DDLT. Smaller grafts were linked to worse graft and patient survival, whereas both small- and large-for-size grafts were associated with longer LOS. Despite these associations, the overall clinical effect of DR size mismatch on outcomes was modest. Thresholds of WR ≤0.74 and BSAR ≤0.85 were associated with only a 6% and 10% increased risk of graft failure, respectively, whereas WR ≤0.65 and BSAR ≤0.85 predicted an 8% and 10% increased risk of death. For LOS, WR ≤0.65 and WR >1.4 corresponded to a 5% and 6% increased risk, whereas BSAR ≤0.78 and BSAR >1.2 were each associated with a 6% increased risk. The absolute effect on LOS was marginal, as the median LOS for both large- and small-for-size grafts was identical to the cohort median of 10 d. We analyzed a small-for-size subgroup to determine additional donor and recipient factors that should be considered when transplanting a small-for-size graft. We also analyzed subpopulations in which size-matching relationships may be confounded. In patients with clinically significant ascites, BSAR ≤0.85 predicted worse outcomes.

Previous studies in adult DDLT have focused on BSAR, as BSA is considered to better correlate with liver volume and metabolism and has been used to estimate standardized liver volume in LDLT.^9,15^ These studies found that both extremes of BSAR correlate with worse graft survival.^12,13,18^ However, studies spanning the most recent decade have found a lesser impact of larger grafts on graft survival and even advocate for increased utilization of large-for-size grafts.^11^ Similarly, we did not find an association between larger graft sizes and worse survival, as well as only a marginal association with prolonged hospitalization. These improvements with larger grafts may be due to improved graft selection or advances in management of large-for-size grafts during the last decade. These strategies typically include parenchymal reduction, delayed abdominal closure, or primary closure with mesh.^19-22^ LFSS is more commonly described among pediatric transplant recipients due to a smaller abdominal domain,^8,23^ and WR has primarily been studied in pediatrics, where adiposity fluctuates less.^24^ Our pediatric DDLT analysis found that WR may serve as a superior metric to BSAR, offering comparable predictive ability while being simpler to calculate; notably, both WR ≤0.70 and WR >2.0 were associated with worse outcomes.^14^ Our current study is the first to assess WR as a predictive size-matching metric in adult DDLT.

ROC analysis showed WR and BSAR had identical area under the curves for 1-y graft failure. Although prior literature favored BSAR, our findings indicate that both BSAR and WR may perform equally well in discriminating risk in most clinical scenarios. Although BSAR can be readily calculated using electronic tools, donor offer evaluation frequently is time-sensitive, and transplant surgeons often evaluate multiple DR combinations during the day. In clinical practice, surgeons often make an initial assessment of size compatibility using donor and recipient weights rather than BSAR. Our findings provide objective support for this commonly used clinical heuristic by demonstrating that WR performs comparably to BSAR in predicting posttransplant outcomes. Given that WR offers a pragmatic advantage in ease of calculation, it may reasonably serve as the default size-matching index, particularly when WR ≤0.74. However, our data also highlight specific contexts in which BSAR provides additional discriminatory value. In multivariable models, both BSAR predicted worse patient survival across 2 lower percentile groups (BSAR ≤0.85), whereas WR <0.65 was predictive only at the extreme. Similarly, BSAR captured risk of ascites up to the 20th percentile (BSAR ≤0.85). This indicates that BSAR captures a broader spectrum of detrimental size mismatches beyond the extremes reflected by WR alone.

Our data suggest that smaller donors have a more significant impact on outcomes than larger donors, particularly when WR ≤0.74 or BSAR ≤0.85. However, we are not advocating an absolute size-mismatch threshold as a contraindication to adult DDLT, especially given the modestly increased risk of graft failure. Rather, DR size mismatch should be considered alongside other donor and recipient factors when evaluating transplant risk. We explored these variables in a subgroup analysis of small-for-size grafts and found that factors related to recipient frailty and poor donor graft quality were associated with worse graft survival. Factors related to recipient frailty include significantly reduced functional status (Karnofsky Performance Status of 10%) and older recipient age. Factors related to poor donor graft quality include controlled DCD and increased donor age.

Additionally, although smaller grafts impacted graft and patient survival, graft discard rates were lower when donors were smaller (lower donor weight and BSA percentile). This is likely because donor body mass index has consistently been cited as an independent predictor of discard, usually due to concerns for hepatic steatosis.^25,26^ Smaller grafts are less likely to harbor obesity-associated comorbidities, skew younger, and are less likely to be biopsied, all contributing to lower discard rates. This finding underscores the importance of careful DR size matching using WR and/or BSAR, as small-for-size grafts are associated with increased risk of graft failure and mortality despite being less frequently discarded. Lower discard rates among smaller donors should not be interpreted as evidence that size mismatch is inconsequential, but rather as indicating that these grafts can be safely used when appropriately matched to the recipient’s dimensions. Allocation policies prioritizing small donors for small recipients have also been postulated to improve the disparity in access to liver transplant among small BSA transplant candidates.^27^

In cases where WR ≤0.74 or BSAR ≤0.85, several strategies may be used to mitigate the risk of SFSS. Undersized grafts create a mismatch in vessel diameter at the site of anastomosis, which can result in turbulent flow, increased vascular resistance, and shunting of arterial flow from the hepatic to splenic artery.^12,28^ These hemodynamic changes reduce graft perfusion and oxygenation, increasing risk of primary nonfunction and acute rejection. Additionally, impaired perfusion limits the delivery of the hepatopoietic factor necessary for graft growth and expansion, preventing it from meeting the metabolic demands of the recipient.^8^ Portal hyperperfusion is another consequence of undersized grafts, and it is associated with poor outcomes, as it causes hepatic congestion, sinusoidal endothelial and Kupffer cell injury with release of inflammatory mediators, and compensatory reductions in arterial inflow.^29-31^ Collectively, these mechanisms underly the pathophysiology of SFSS. To avoid this cascade, surgical management involves 2 complementary strategies: optimizing venous outflow to prevent hepatic congestion and limiting inflow to reduce hyperperfusion. Inflow modulation strategies include splenic artery ligation to both improve hepatic arterial inflow and reduce portal venous pressure,^32^ whereas hemiportocaval shunting reduces hyperperfusion by diverting excess portal flow to the systemic vasculature.^33^ Outflow modulation strategies in the context of DDLT focus on maximizing the dimensions of the caval anastomosis, with techniques including traditional caval replacement, “piggyback” end-to-side anastomoses, and side-to-side cavocavostomy. Caval replacement involves removal of the recipient retrohepatic inferior vena cava (IVC) and replacement with a donor IVC, whereas “piggyback” involves preservation of the recipient IVC and ligation of the recipient short hepatic veins.^34^ Finally, side-to-side cavocavostomy involves a longitudinal anastomosis between the donor and recipient IVC walls. Although reported studies have found no significant differences in rates of hepatic outflow obstruction, side-to-side cavocavostomy is postulated to create the widest outflow tract and therefore may be most advantageous for small-for-size grafts.^35,36^

BSAR ≤0.85 was predictive of worse outcomes in patients with at least moderate ascites. Body weight in end-stage liver disease is highly variable due to fluid shifts from ascites and edema,^6^ and substantial abdominal distention can falsely elevate recipient weight at the time of transplant, thereby misrepresenting true abdominal domain. Because BSA is less sensitive to these fluctuations, BSAR may serve as a more reliable metric in this population.

Traditionally, the effect of size mismatch on transplant outcomes has been shown to intensify at both extremes, resulting in a characteristic “U-shaped” relationship. This nonlinearity limits the utility of conventional statistics, such as modeling size indices as continuous variables in Cox regression. In our exploratory analysis, Martingale residuals confirmed that size-matching indices such as WR and BSAR violate the linearity assumption of the Cox proportional hazards model, supporting the need for alternative analytic strategies. To address this, we stratified patients into equal percentile groups such that each group had sufficient sample size to detect clinical associations at both ends of the size-mismatch spectrum. However, prior studies have successfully estimated continuous HRs using generalized additive models with smoothing splines, which accommodate such nonlinear associations.^18,37^ Future studies using these advanced methods will be essential to define precise thresholds of size mismatch for both WR and BSAR.

This study is limited by its retrospective design, which may introduce unmeasured bias and restrict causal inference. The UNOS database is subject to variability in data entry and only captures broad outcomes, preventing evaluation of more specific, clinically relevant complications. Center-specific factors are not accounted for, which can limit generalizability, and there is no standardized surgical management for DDLT. We conducted multiple comparisons across endpoints, indices (WR and BSAR), subgroup analyses, and threshold explorations without adjustment for multiplicity, which may increase the risk of type I error. Because WR and BSAR are highly correlated measures of DR size matching, formal correction methods may also increase the risk of type II error. Furthermore, fluid excess is practically universal in end-stage liver disease and can lead to inaccurate size-matching ratios. Hepatic steatosis is an independent risk factor for graft failure and SFSS that cannot be controlled for, as UNOS lacks sufficient diagnostic codes for steatosis. The DR WR and BSAR cutoffs used in this study were selected by the percentile, and it is possible that the true threshold for clinically significant size mismatch lies at higher or lower ratios. Generalized additive models that account for nonlinear associations should be used to determine more exact WR and BSAR cutoffs. External validation will be important to confirm the generalizability of our findings, both for the predictive performance of WR and BSAR demonstrated in ROC analysis and for their observed associations with graft and patient outcomes, as these results may otherwise reflect sample-specific effects.

In adult whole-liver transplant, only lower DR size matching ratios increase risk of graft failure and death, while both extremes marginally prolong LOS. Although WR can be considered the *de facto* size-matching metric in adult DDLT, BSAR should be preferred in patients with at least moderate ascites. Transplant surgeons should exercise caution if they encounter a WR ≤0.74 or a BSAR ≤0.85.

## Supplementary Material


