# Supplementary material for: The Impact of Donor-Recipient Weight Ratios and Body Surface Area Ratios on Outcomes After Adult Deceased Donor Whole-Liver Transplantation
Source: Transplant Direct. 2026 Aug 21;12(9):e1991. doi: 10.1097/TXD.0000000000001991 (PMC13502760; doi:10.1097/TXD.0000000000001991)

SDC, Table 1: Risk factors considered in univariable analysis. Hazard ratios and p-values are calculated for graft survival. Variables were included in multivariable model if p < 0.20.

Variables	HR (95% CI)	P-value
ABO incompatibility	1.11 (0.96, 1.28)	**0.14**
ABO compatibility	1.06 (0.99, 1.14)	**0.09**
Recipient age 18-30 years	0.94 (0.85, 1.03)	**0.19**
Recipient age 60-65 years	1.09 (1.05, 1.14)	**<0.001**
Recipient age ≥ 65 years	1.43 (1.38, 1.49)	**<0.001**
Donor age ≤ 10 years	0.91 (0.71, 1.16)	0.44
Donor age 10-15 years	0.83 (0.71, 0.97)	**0.02**
Donor age 15-20 years	0.90 (0.84, 0.96)	**0.002**
Donor age 20-30 years	0.89 (0.85, 0.93)	**<0.001**
Donor age 45-55 years	1.02 (0.98, 1.06)	0.35
Donor age 55-60 years	1.10 (1.04, 1.16)	**<0.001**
Donor age 60-70 years	1.19 (1.13, 1.25)	**<0.001**
Donor age ≥ 70 years	1.30 (1.20, 1.40)	**<0.001**
Recipient albumin 1.5-2.0 mg/dL	1.06 (0.97, 1.14)	**0.19**
Recipient albumin ≤ 1.5 mg/dL	0.95 (0.76, 1.18)	0.63
Recipient albumin 2.0-2.5 mg/dL	1.00 (0.96, 1.05)	0.93
Ascites	1.03 (0.99, 1.07)	**0.11**
Spontaneous bacterial peritonitis	0.93 (0.87, 0.98)	**0.01**

Recipient BMI 30-35 kg/m^2	1.01 (0.98, 1.05)	0.48
Recipient BMI 35-40 kg/m^2	1.01 (0.96, 1.07)	0.61
Recipient BMI ≥ 40 kg/m^2	1.09 (1.01, 1.17)	**0.02**
Cold ischemia time ≤ 6 hours	0.92 (0.89, 0.96)	**<0.001**
Cold ischemia time 12-14 hours	1.03 (0.88, 1.22)	0.68
Cold ischemia time ≥ 14 hours	1.18 (1.02, 1.38)	**0.03**
Controlled donation after circulatory death	1.21 (1.14, 1.28)	**<0.001**
Donor creatinine 1.5-2.0 mg/dL	1.08 (1.03, 1.14)	**0.004**
Donor creatinine ≥ 2.0 mg/dL	1.07 (1.03, 1.11)	**0.001**
Hepatitis C cirrhosis	0.93 (0.89, 0.98)	**0.005**
Hemodialysis	1.27 (1.21, 1.33)	**<0.001**
Transplant center distance 500-1,000 miles	1.07 (0.99, 1.15)	**0.11**
Transplant center distance ≥ 1,000 miles	0.96 (0.83, 1.10)	0.54
Drop out	1.02 (0.95, 1.10)	0.57
High school	1.10 (1.06, 1.13)	**<0.001**
Technical school	0.99 (0.95, 1.02)	0.47
Bachelor’s degree	0.90 (0.87, 0.95)	**<0.001**
Doctorate	0.91 (0.85, 0.97)	**0.005**

Hepatic encephalopathy	1.22 (1.17, 1.28)	**<0.001**
African American recipient	1.21 (1.15, 1.29)	**<0.001**
African American donor	1.07 (1.02, 1.12)	**0.003**
Hepatocellular carcinoma	1.09 (1.04, 1.13)	**<0.001**
MELD score 30-35	0.97 (0.92, 1.02)	0.21
MELD score 35-40	0.98 (0.93, 1.04)	0.51
MELD score ≥ 40	1.15 (1.09, 1.21)	**<0.001**
Serum sodium 130-135 mEq/L	0.98 (0.95, 1.02)	0.42
Serum sodium 125-130 mEq/L	0.87 (0.82, 0.92)	**<0.001**
Serum sodium ≤ 120 mEq/L	0.86 (0.78, 0.94)	**0.001**
Serum sodium 145-150 mEq/L	1.23 (1.11, 1.36)	**<0.001**
Serum sodium 150-155 mEq/L	1.40 (1.13, 1.73)	**0.002**
Serum sodium 155-160 mEq/L	1.90 (1.21, 2.98)	**0.005**
Serum sodium ≥ 161 mEq/L	1.68 (0.70, 4.04)	0.25
KPS functional status 10%	1.34 (1.25, 1.43)	**<0.001**
KPS functional status 20%	1.14 (1.09, 1.20)	**<0.001**
KPS functional status 50%	1.04 (0.99, 1.09)	**0.10**
KPS functional status 60%	0.94 (0.89, 0.99)	**0.02**
KPS functional status 70%	0.95 (0.91, 1.00)	**0.04**

KPS functional status 80%	0.86 (0.82, 0.91)	**<0.001**
KPS functional status 90%	0.77 (0.70, 0.85)	**<0.001**
KPS functional status 100%	0.89 (0.73, 1.08)	0.23
HCV-positive serology (donor)	1.02 (0.96, 1.08)	0.58
INR 2.0-2.5	0.97 (0.92, 1.01)	**0.17**
INR 2.5-3.0	0.96 (0.90, 1.02)	**0.15**
INR 3.0-3.5	0.92 (0.85, 0.99)	**0.02**
INR 3.5-4.0	0.92 (0.82, 1.04)	**0.18**
INR ≥ 4.0	0.88 (0.80, 0.97)	**0.007**
Life support	1.34 (1.27, 1.42)	**<0.001**
ICU admission	1.25 (1.19, 1.31)	**<0.001**
Hospital admission	0.95 (0.91, 0.99)	**0.02**
Donor pH 7.1-7.2	1.08 (0.91, 1.29)	0.39
Donor pH 7.0-7.1	0.91 (0.58, 1.43)	0.68
Donor pH 7< 7.0	0.78 (0.35, 1.74)	0.55
History of portal vein thrombosis	1.18 (1.11, 1.26)	**<0.001**
Private health insurance	0.78 (0.75, 0.81)	**<0.001**
Medicaid	0.99 (0.95, 1.04)	0.73
Previous upper abdominal surgery	1.11 (1.07, 1.14)	**<0.001**
Region 1	1.13 (1.04, 1.23)	**0.003**
Region 2	1.13 (1.08, 1.19)	**<0.001**

Region 3	0.95 (0.91, 1.00)	**0.04**
Region 4	1.05 (1.00, 1.11)	**0.06**
Region 5	0.96 (0.91, 1.01)	**0.09**
Region 6	0.75 (0.67, 0.83)	**<0.001**
Region 7	1.00 (0.94, 1.06)	0.95
Region 8	0.97 (0.91, 1.04)	0.39
Region 9	1.00 (0.92, 1.08)	0.96
Region 10	0.96 (0.91, 1.02)	**0.18**
Region 11	1.03 (0.98, 1.09)	0.21
Donor AST 90-140 IU/L	0.97 (0.92, 1.03)	0.39
Donor AST ≥ 140 IU/L	1.00 (0.95, 1.05)	0.96
Donor ALT 60-100 IU/L	0.97 (0.92, 1.02)	0.20
Donor ALT ≥ 100 IU/L	0.95 (0.91, 0.99)	**0.02**
Regional allocation	1.02 (0.98, 1.06)	0.37
National allocation	1.12 (1.06, 1.18)	**<0.001**
Recipient total bilirubin ≤ 2 mg/dL	1.15 (1.11, 1.19)	**<0.001**
Recipient total bilirubin 8-16 mg/dL	0.92 (0.88, 0.97)	**0.001**
Recipient total bilirubin 16-32 mg/dL	1.00 (0.95, 1.05)	0.86
Recipient total bilirubin ≥ 33 mg/dL	1.02 (0.96, 1.10)	0.49
TIPS procedure	1.12 (1.06, 1.18)	**<0.001**

HR, Hazard ratio; CI, Confidence interval; KPS, Karnofsky Performance Status; HCV, Hepatitis C virus; AST, Aspartate aminotransferase; ALT, Alanine aminotransferase; TIPS, Transjugular intrahepatic portosystemic shunt
Bold numbers indicate P-value < 0.20

Supplementary Table 2: Graft discard rates by donor weight (kg) and body surface area (m^2).

	**Donor weight ≤ 56.4 (10th percentile)**	**Donor weight 56.4 < weight ≤ 65 (10th-20th percentile)**	**Donor weight 65< weight ≤ 102.3 (20th-80th percentile)**	**Donor weight 102.3< weight ≤ 115 (80th-90th percentile)**	**Donor weight > 115 (90th percentile)**	**P-value**
n	11,167	11,133	66,699	11,138	11,236	
Graft Discard	678 (6.1%)	744 (6.7%)	4,909 (7.4%)	1,046 (9.4%)	1,154 (10.3%)	**<0.001**
	**Donor BSA ≤ 1.60 (10th percentile)**	**Donor BSA 1.60 < BSA ≤ 1.73 (10th-20th percentile)**	**Donor BSA 1.73< BSA ≤ 2.23 (20th-80th percentile)**	**Donor BSA 2.23 < WR ≤ 2.38 (80th-90th percentile)**	**Donor BSA > 2.38 (90th percentile)**	**P-value**
n	11,117	11,113	66,723	11,101	11,319	
Graft Discard	686 (6.2%)	800 (7.2%)	4,899 (7.3%)	999 (9.0%)	1,147 (10.1%)	**<0.001**

SDC, Table 3: Multivariable model results for patient survival in all adult patients receiving DDLT.

Variables	HR (95% CI)	P-value
WR Percentiles		
Bottom 10th percentile (WR ≤ 0.65)	1.08 (1.01, 1.15)	**0.02**
10th-20th percentile (0.65 < WR ≤ 0.74)	1.04 (0.98, 1.10)	0.21
20th-80th percentile (0.74 < WR ≤ 1.2)	Ref	
80th-90th percentile (1.2 < WR ≤ 1.4)	1.00 (0.94, 1.06)	0.93
Upper 90th percentile (WR > 1.4)	1.03 (0.97, 1.10)	0.30
BSAR Percentiles		
Bottom 10th percentile (BSAR ≤ 0.78)	1.14 (1.08, 1.21)	**<0.001**
10th-20th percentile (0.78 < BSAR ≤ 0.85)	1.10 (1.04, 1.16)	**0.001**
20th-80th percentile (0.85 < BSAR ≤ 1.1)	Ref	
80th-90th percentile (1.1 < BSAR ≤ 1.2)	1.00 (0.94, 1.06)	0.96
Upper 90th percentile (BSAR > 1.2)	0.99 (0.94, 1.05)	0.78
Recipient age 18-30 years	0.81 (0.72, 0.92)	**<0.001**
Recipient age 60-65 years	1.37 (1.31, 1.44)	**<0.001**

Recipient age ≥ 65 years	1.66 (1.58, 1.74)	**<0.001**
Donor age ≤ 10 years	0.75 (0.56, 1.01)	0.06
Donor age 10-15 years	0.87 (0.73, 1.03)	0.10
Donor age 15-20 years	0.97 (0.90, 1.04)	0.38
Donor age 20-30 years	0.96 (0.92, 1.01)	0.15
Donor age 55-60 years	1.12 (1.05, 1.19)	**<0.001**
Donor age 60-70 years	1.16 (1.10, 1.23)	**<0.001**
Donor age ≥ 70 years	1.21 (1.11, 1.32)	**<0.001**
Recipient Albumin ≤ 1.5 mg/dL	0.90 (0.71, 1.15)	0.41
Ascites	1.07 (1.02, 1.12)	**0.01**
Spontaneous bacterial peritonitis	0.95 (0.89, 1.02)	0.13
Recipient BMI 35-40 kg/m^2	1.03 (0.97, 1.09)	0.36
Recipient BMI ≥ 40 kg/m^2	1.07 (0.98, 1.16)	0.12
Cold ischemia time ≤ 6 hours	0.93 (0.90, 0.97)	**<0.001**
Cold ischemia time ≥ 14 hours	1.23 (1.04, 1.46)	**0.01**
Donor creatinine 1.5-2.0 mg/dL	1.08 (1.02, 1.15)	**0.01**
Donor creatinine ≥ 2.0 mg/dL	1.08 (1.03, 1.12)	**<0.001**
Hepatitis C cirrhosis	0.93 (0.88, 0.98)	**0.01**
Hemodialysis	1.20 (1.11, 1.28)	**<0.001**
Drop out	0.96 (0.84, 1.09)	0.49
High school	1.03 (0.93, 1.15)	0.57

Technical school	0.95 (0.85, 1.06)	0.38
Bachelor's degree	0.89 (0.80, 1.00)	**0.047**
Doctorate	0.85 (0.75, 0.96)	**0.01**
Hepatic encephalopathy	1.15 (1.09, 1.22)	**<0.001**
African American recipient	1.16 (1.09, 1.24)	**<0.001**
African American donor	1.03 (0.98, 1.08)	0.20
Hepatocellular carcinoma	1.07 (1.02, 1.13)	**0.01**
MELD score ≥ 40	1.08 (1.00, 1.17)	**0.047**
Serum sodium 125-130 mEq/L	0.97 (0.91, 1.03)	0.33
Serum sodium ≤ 125 mEq/L	0.97 (0.88, 1.07)	0.50
Serum sodium 145-150 mEq/L	1.08 (0.97, 1.21)	0.15
Serum sodium 150-155 mEq/L	1.30 (1.04, 1.61)	**0.02**
Serum sodium ≥ 160 mEq/L	1.83 (0.76, 4.41)	0.18
KPS functional status 10%	1.14 (1.02, 1.26)	**0.02**
KPS functional status 20%	1.08 (1.01, 1.15)	**0.02**
KPS functional status 50%	0.96 (0.91, 1.02)	0.25
KPS functional status 60%	0.83 (0.78, 0.89)	**<0.001**
KPS functional status 70%	0.83 (0.78, 0.88)	**<0.001**
KPS functional status 80%	0.78 (0.73, 0.84)	**<0.001**
KPS functional status 90%	0.67 (0.60, 0.75)	**<0.001**
KPS functional status 100%	0.84 (0.68, 1.03)	0.09
HCV-positive serology (donor)	1.13 (1.05, 1.20)	**<0.001**

INR 2.0-2.5	0.95 (0.90, 1.00)	**0.049**
INR 3.0-3.5	0.88 (0.81, 0.96)	**<0.001**
INR 3.5-4.0	0.89 (0.78, 1.01)	0.08
INR ≥ 4.0 2	0.79 (0.71, 0.88)	**<0.001**
Life support	1.14 (1.05, 1.24)	**<0.001**
ICU admission	1.07 (0.99, 1.15)	0.09
Donor pH 7.1-7.2	1.12 (0.94, 1.34)	0.20
History of portal vein thrombosis	1.14 (1.07, 1.22)	**<0.001**
Private health insurance	0.84 (0.81, 0.87)	**<0.001**
Previous upper abdominal surgery	1.06 (1.02, 1.10)	**<0.001**
Region 1	1.12 (1.03, 1.23)	**0.01**
Region 2	1.16 (1.10, 1.23)	**<0.001**
Region 4	1.06 (0.99, 1.12)	0.09
Region 5	0.89 (0.84, 0.94)	**<0.001**
Region 6	0.87 (0.77, 0.98)	**0.02**
Region 8	1.01 (0.93, 1.09)	0.83
Region 11	1.09 (1.03, 1.16)	**0.01**
Donor AST 90-140 IU/L	0.96 (0.90, 1.02)	0.20
Donor AST 60-100 IU/L	0.98 (0.93, 1.03)	0.41
National allocation	1.08 (1.02, 1.15)	**0.01**
Recipient total bilirubin ≤ 2 mg/dL	1.21 (1.15, 1.27)	**<0.001**

Recipient total bilirubin 8-16 mg/dL	0.99 (0.94, 1.05)	0.81
Recipient total bilirubin 16-32 mg/dL	1.01 (0.95, 1.07)	0.80
TIPS procedure	1.12 (1.06, 1.19)	**<0.001**

HR, Hazard ratio; CI, Confidence interval; KPS, Karnofsky Performance Status; HCV, Hepatitis C virus; AST, Aspartate aminotransferase; ALT, Alanine aminotransferase; TIPS, Transjugular intrahepatic portosystemic shunt

Bold numbers indicate P-value<0.20

SDC, Table 4: Multivariable model results for LOS in all adult patients receiving DDLT.

Variables	HR (95% CI)	P-Value
WR Percentiles		
Bottom 10th percentile (WR ≤ 0.65)	0.95 (0.92, 0.97)	**<0.001**
10th-20th percentile (0.65 < WR ≤ 0.74)	1.01 (0.99, 1.04)	0.32
20th-80th percentile (0.74 < WR ≤ 1.2)	Ref	
80th-90th percentile (1.2 < WR ≤ 1.4)	0.99 (0.97, 1.02)	0.56
Upper 90th percentile (WR > 1.4)	0.94 (0.92, 0.97)	**<0.001**
BSAR Percentiles		
Bottom 10th percentile (BSAR ≤ 0.78)	0.94 (0.92, 0.97)	**<0.001**
10th-20th percentile (0.78 < BSAR ≤ 0.85)	1.01 (0.99, 1.04)	0.27
20th-80th percentile (0.85 < BSAR ≤ 1.1)	Ref	
80th-90th percentile (1.1 < BSAR ≤ 1.2)	0.98 (0.95, 1.00)	0.11
Upper 90th percentile (BSAR > 1.2)	0.94 (0.91, 0.96)	**<0.001**
ABO incompatibility	0.90 (0.85, 0.97)	**<0.001**
ABO compatibility	0.99 (0.96, 1.02)	0.58
Recipient age 18-30 years	1.02 (0.98, 1.07)	0.34

Recipient age 60-65 years	0.91 (0.89, 0.93)	**<0.001**
Recipient age ≥ 65 years	0.90 (0.88, 0.92)	**<0.001**
Donor age ≤ 10 years	1.02 (0.91, 1.14)	0.71
Donor age 10-15 years	1.00 (0.94, 1.08)	0.94
Donor age 15-20 years	1.01 (0.97, 1.04)	0.70
Donor age 20-30 years	0.99 (0.97, 1.02)	0.62
Donor age 45-55 years	0.99 (0.96, 1.01)	0.21
Donor age 55-60 years	0.97 (0.95, 1.00)	0.07
Donor age 60-70 years	0.98 (0.95, 1.00)	0.09
Donor age ≥ 70 years	1.00 (0.96, 1.04)	0.91
Recipient Albumin 2.0-2.5 mg/dL	0.95 (0.93, 0.97)	**<0.001**
Recipient Albumin 1.5-2.0 mg/dL	0.92 (0.88, 0.96)	**<0.001**
Recipient Albumin ≤ 1.5 mg/dL	0.88 (0.80, 0.97)	**0.01**
Ascites	0.97 (0.95, 0.99)	**<0.001**
Spontaneous bacterial peritonitis	0.99 (0.97, 1.02)	0.61
Recipient BMI 30-35 kg/m^2	0.98 (0.96, 1.00)	**0.04**
Recipient BMI 35-40 kg/m^2	0.95 (0.92, 0.97)	**<0.001**
Recipient BMI ≥ 40 kg/m^2	0.91 (0.88, 0.94)	**<0.001**
Cold ischemia time ≤ 6 hours	1.11 (1.09, 1.13)	**<0.001**
Cold ischemia time ≥ 14 hours	1.01 (0.96, 1.06)	0.79

Controlled donation after circulatory death	1.03 (1.01, 1.06)	**0.01**
Donor creatinine ≥ 2.0 mg/dL	0.98 (0.96, 1.00)	**0.04**
Hepatitis C cirrhosis	0.98 (0.96, 1.01)	0.17
Hemodialysis	0.79 (0.76, 0.81)	**<0.001**
Distance 500-1,000 miles	1.03 (0.99, 1.07)	0.13
Distance ≥ 1,000 miles	1.03 (0.96, 1.10)	0.45
Drop out	0.95 (0.91, 0.98)	**<0.001**
Bachelor's degree	1.00 (0.98, 1.02)	0.71
Hepatic encephalopathy	0.88 (0.86, 0.90)	**<0.001**
African American recipient	0.94 (0.91, 0.96)	**<0.001**
Hepatocellular carcinoma	1.15 (1.12, 1.18)	**<0.001**
MELD score 3-35	0.93 (0.90, 0.95)	**<0.001**
MELD score 35-40	0.89 (0.86, 0.93)	**<0.001**
MELD score ≥ 40	0.87 (0.83, 0.92)	**<0.001**
Serum sodium ≤ 125 mEq/L	0.94 (0.91, 0.98)	**<0.001**
Serum sodium 145-150 mEq/L	0.88 (0.83, 0.92)	**<0.001**
Serum sodium 150-155 mEq/L	0.81 (0.72, 0.91)	**<0.001**
Serum sodium 155-160 mEq/L	0.84 (0.65, 1.09)	0.20
Serum sodium ≥ 160 mEq/L	0.93 (0.57, 1.51)	0.76

KPS functional status 10%	0.99 (0.95, 1.04)	0.74
KPS functional status 20%	0.96 (0.94, 0.99)	**0.01**
KPS functional status 50%	1.03 (1.00, 1.06)	**0.03**
KPS functional status 60%	1.15 (1.12, 1.18)	**<0.001**
KPS functional status 70%	1.21 (1.18, 1.25)	**<0.001**
KPS functional status 80%	1.28 (1.25, 1.32)	**<0.001**
KPS functional status 90%	1.21 (1.16, 1.26)	**<0.001**
KPS functional status 100%	1.34 (1.23, 1.45)	**<0.001**
HCV-positive serology (donor)	1.05 (1.03, 1.09)	**<0.001**
INR 2.0-2.5	0.96 (0.94, 0.99)	**<0.001**
INR 2.5-3.0	0.98 (0.95, 1.00)	0.09
INR 3.0-3.5	0.99 (0.95, 1.02)	0.49
INR 3.5-4.0	1.00 (0.94, 1.05)	0.91
INR $\geq$ 4.0	1.07 (1.02, 1.12)	**<0.001**
Life support	0.87 (0.84, 0.91)	**<0.001**
ICU admission	0.70 (0.67, 0.72)	**<0.001**
Hospital admission	0.80 (0.78, 0.82)	**<0.001**
History of portal vein thrombosis	0.93 (0.91, 0.96)	**<0.001**
Private health insurance	1.13 (1.11, 1.15)	**<0.001**
Medicaid	1.01 (0.98, 1.03)	0.55
Previous upper abdominal surgery	0.97 (0.95, 0.98)	**<0.001**

Region 1	0.89 (0.86, 0.93)	**<0.001**
Region 2	0.83 (0.80, 0.85)	**<0.001**
Region 3	1.04 (1.02, 1.07)	**<0.001**
Region 5	1.01 (0.99, 1.04)	0.30
Region 6	1.07 (1.02, 1.12)	**0.01**
Region 8	0.93 (0.90, 0.96)	**<0.001**
Region 9	0.71 (0.68, 0.73)	**<0.001**
Region 10	0.97 (0.94, 1.00)	0.06
Region 11	1.11 (1.08, 1.14)	**<0.001**
Donor AST ≥ 140 IU/L	0.99 (0.97, 1.02)	0.57
Donor ALT ≥ 100 IU/L	1.03 (1.01, 1.05)	**0.01**
Regional allocation	1.01 (0.99, 1.03)	0.18
National allocation	1.00 (0.97, 1.02)	0.69
Recipient total bilirubin 8-16 mg/dL	0.97 (0.95, 1.00)	**0.047**
Recipient total bilirubin ≥ 16-32 mg/dL	0.97 (0.94, 1.00)	**0.046**
Recipient total bilirubin ≥ 32 mg/dL	0.93 (0.89, 0.96)	**<0.001**
TIPS procedure	0.93 (0.91, 0.96)	**<0.001**

HR, Hazard ratio; CI, Confidence interval; KPS, Karnofsky Performance Status; HCV, Hepatitis C virus; AST, Aspartate aminotransferase; ALT, Alanine aminotransferase; TIPS, Transjugular intrahepatic portosystemic shunt

SDC, Figure 1A: Kaplan-Meier estimate of graft survival in patients with acute alcoholic hepatitis stratified by <10th weight ratio percentile, 20th-80th weight ratio percentile (reference), and >90th weight raito percentile.

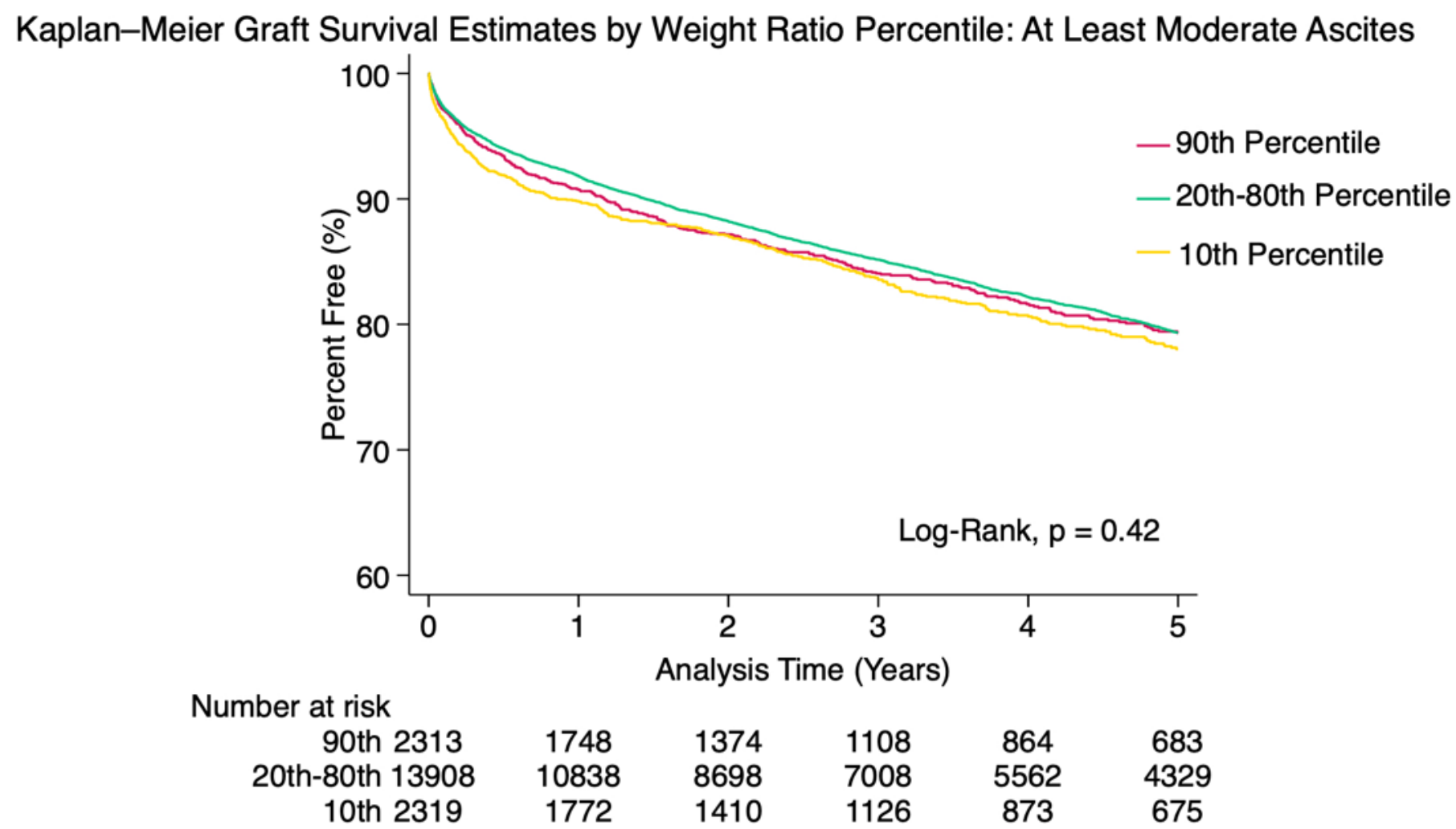

SDC, Figure 1B: Kaplan-Meier estimate of graft survival in patients with acute alcoholic hepatitis stratified by <10th body surface area ratio percentile, 20th-80th body surface area ratio percentile (reference), and >90th body surface area percentile.

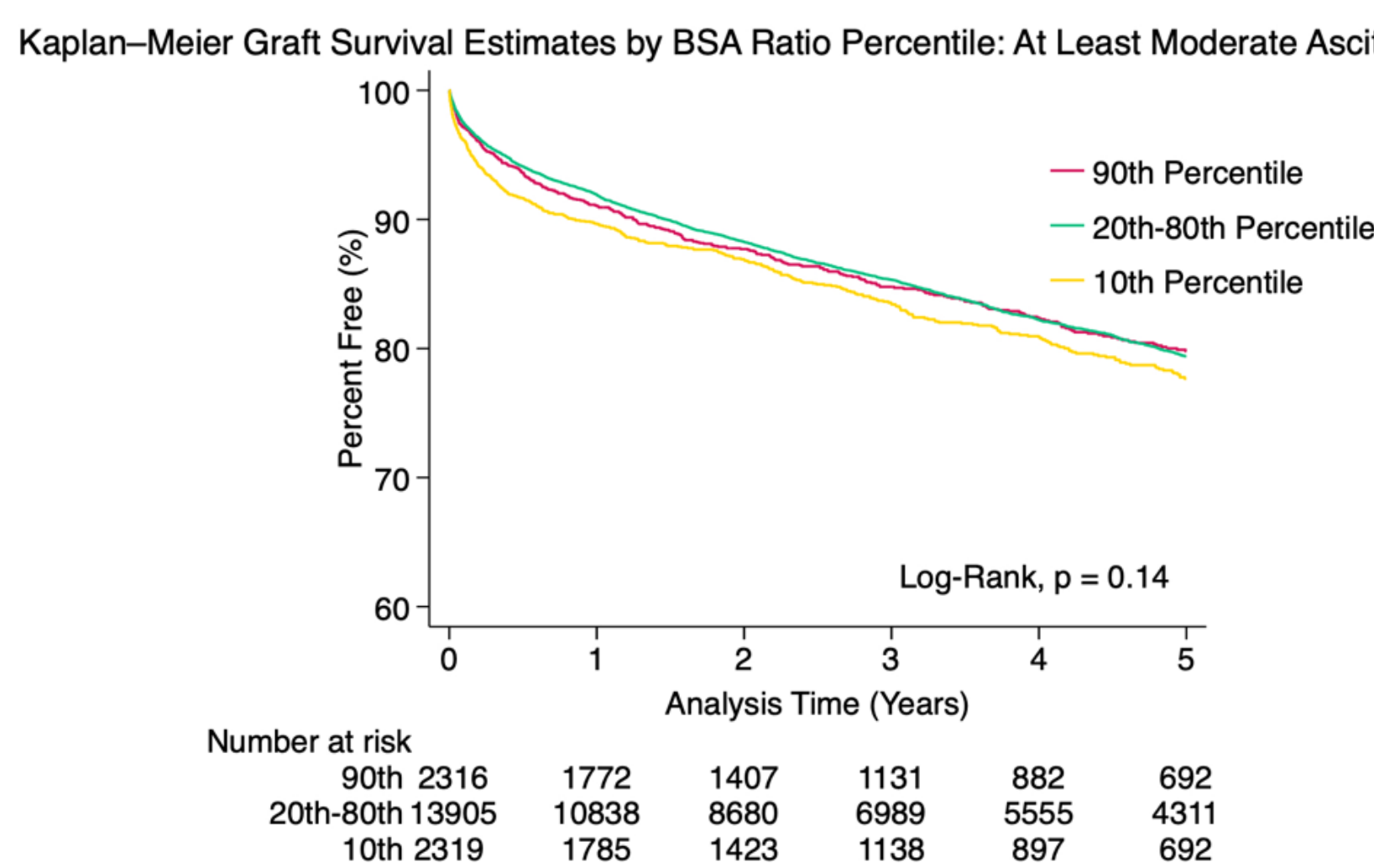

SDC, Figure 2A-B: Martingale residual plots with locally weighted scatterplot smoothing curves evaluating whether (A) weight ratio or (B) body surface area ratio violates the linearity assumption of the Cox proportional hazards model.

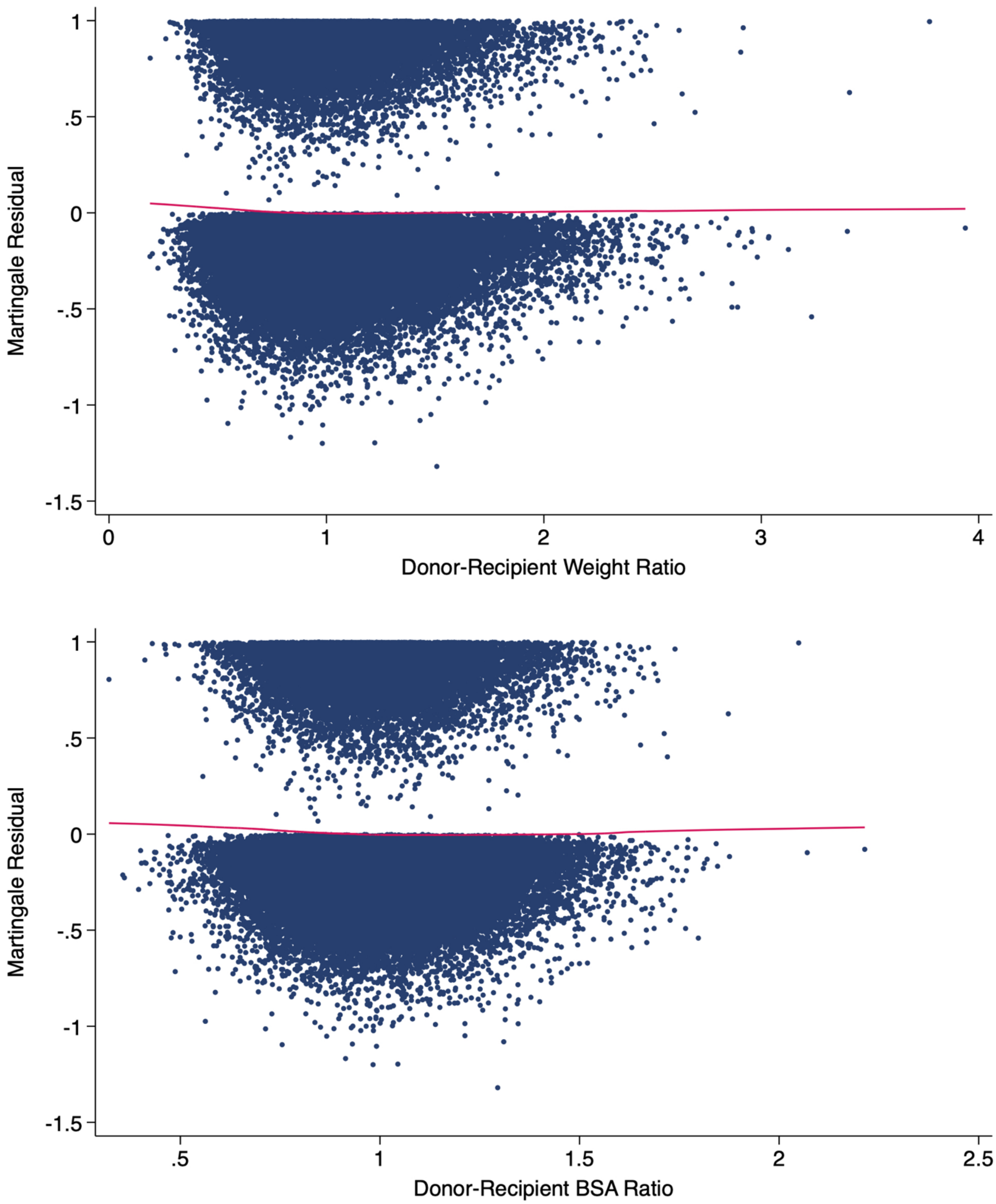

Supplement: Supplementary file 1 [file txd-12-e1991-s001.pdf]
